# National action plans for antimicrobial resistance and variations in surveillance data platforms

**DOI:** 10.2471/BLT.22.289403

**Published:** 2023-05-29

**Authors:** Scott JC Pallett, Esmita Charani, Lois Hawkins, Andrea Mazzella, Vanesa Anton-Vazquez, Rishi Banerjee, Terry J Evans, Benjamin Patterson, Sathyavani Subbarao, Saleh Alqahtani, Marina Basarab, Aodhan S Breathnach, Nabeela Mughal, Luke SP Moore

**Affiliations:** a Centre of Defence Pathology, Royal Centre for Defence Medicine, Queen Elizabeth Hospital Birmingham, Birmingham, B15 2WB, England.; b Centre of Excellence in Infectious Diseases Research, University of Liverpool, Liverpool, England.; c Infection and Immunity Clinical Academic Group, St George’s University Hospitals NHS Foundation Trust, London, England.; d Institute for Infection and Immunity, St George’s University of London, London, England.; e Medicine at Sibley Memorial Hospital, Johns Hopkins University, Baltimore, United States of America.; f Clinical Infection Department, Chelsea and Westminster Hospital, London, England.; g National Institute for Health Research Health Protection Research Unit in Healthcare Associated Infections and Antimicrobial Resistance, Imperial College London, London, England.

## Abstract

**Objective:**

To assess how national antimicrobial susceptibility data used to inform national action plans vary across surveillance platforms.

**Methods:**

We identified available open-access, supranational, interactive surveillance platforms and cross-checked their data in accordance with the World Health Organization’s (WHO’s) Data Quality Assurance: module 1. We compared platform usability and completeness of time-matched data on the antimicrobial susceptibilities of four blood isolate species: *Escherichia coli, Klebsiella pneumoniae*, *Staphylococcus aureus* and *Streptococcus pneumoniae* from WHO’s Global Antimicrobial Resistance and Use Surveillance System, European Centre for Disease Control’s (ECDC’s) network and Pfizer’s Antimicrobial Testing Leadership and Surveillance database. Using Bland–Altman analysis, paired *t*-tests, and Wilcoxon signed-rank tests, we assessed susceptibility data and number of isolate concordances between platforms.

**Findings:**

Of 71 countries actively submitting data to WHO, 28 also submit to Pfizer’s database; 19 to ECDC; and 16 to all three platforms. Limits of agreement between WHO’s and Pfizer’s platforms for organism–country susceptibility data ranged from −26% to 35%. While mean susceptibilities of WHO’s and ECDC‘s platforms did not differ (bias: 0%, 95% confidence interval: −2 to 2), concordance between organism–country susceptibility was low (limits of agreement −18% to 18%). Significant differences exist in isolate numbers reported between WHO–Pfizer (mean of difference: 674, *P*-value: < 0.001, and WHO–ECDC (mean of difference: 192, *P*-value: 0.04) platforms.

**Conclusion:**

The considerable heterogeneity of nationally submitted data to commonly used antimicrobial resistance surveillance platforms compromises their validity, thus undermining local and global antimicrobial resistance strategies. Hence, we need to understand and address surveillance platform variability and its underlying mechanisms.

## Résumé

**Objectif:**

Évaluer de quelle manière les données nationales relatives à la sensibilité aux antimicrobiens, qui servent à établir des plans d'action nationaux, varient d'une plateforme de surveillance à l'autre.

**Méthodes:**

Nous avons identifié les plateformes de surveillance interactives, supranationales et en libre accès, et avons recoupé leurs données conformément au module 1 du Contrôle de la qualité des données de l'Organisation mondiale de la Santé (OMS). Nous avons comparé la facilité d'utilisation et l'exhaustivité des informations synchronisées concernant la sensibilité aux antimicrobiens pour quatre types d'isolats d'hémoculture: *Escherichia coli, Klebsiella pneumoniae*, *Staphylococcus aureus* et *Streptococcus pneumoniae*, issus du Système mondial de surveillance de la résistance aux antimicrobiens de l'OMS, du réseau du Centre européen de prévention et de contrôle des maladies (ECDC) ainsi que de la base de données du Programme de surveillance de la résistance aux antimicrobiens (ATLAS) de Pfizer. À l'aide d'une analyse de Bland-Altman, de tests *t* jumelés et de tests des rangs signés de Wilcoxon, nous avons évalué les données relatives à la sensibilité et le nombre d'isolats concordants entre les plateformes.

**Résultats:**

Sur 71 pays qui transmettent activement leurs données à l'OMS, 28 les fournissent également à la base de données de Pfizer; 19 à l'ECDC; et 16 à l'ensemble des trois plateformes. Les limites de l'accord entre les plateformes de l'OMS et de Pfizer pour les données de sensibilité par organisme–pays étaient comprises entre −26% et 35%. Bien que la sensibilité moyenne des plateformes de l'OMS et de l'ECDC ne présente aucune différence (biais: 0%, intervalle de confiance de 95%: −2 à 2), la concordance en termes de sensibilité par organisme–pays était faible (limites de l'accord comprises entre −18 et 18%). Des variations considérables existent au niveau du nombre d'isolats entre les plateformes de l'OMS et de Pfizer (différence moyenne: 674, valeur p: < 0,001) et entre celles de l'OMS et de l'ECDC (différence moyenne: 192, valeur p: 0,04).

**Conclusion:**

La grande hétérogénéité des données soumises par les pays à des plateformes de surveillance de la résistance aux antimicrobiens couramment utilisées compromet leur validité, et par conséquent les stratégies locales et mondiales de lutte contre cette résistance. Nous devons donc comprendre et résoudre ce manque de régularité des plateformes de surveillance ainsi que ses mécanismes sous-jacents.

## Resumen

**Objetivo:**

Analizar cómo difieren los datos nacionales sobre susceptibilidad antimicrobiana utilizados para conformar los planes nacionales de acción, entre las diferentes plataformas de vigilancia.

**Métodos Se i:**

dentificaron las plataformas de vigilancia disponibles, de libre acceso, supranacionales e interactivas, y se llevó a cabo una comprobación cruzada de sus datos, de conformidad con el Control de calidad de datos: módulo 1 de la Organización Mundial de la Salud (OMS). Se realizó una comparación entre la utilidad de la plataforma y la exhaustividad de los datos, coincidentes en el tiempo y relativos a la susceptibilidad antimicrobiana de cuatro tipos de bacterias sanguíneas aisladas: *Escherichia coli, Klebsiella pneumoniae*, *Staphylococcus aureus* y *Streptococcus pneumoniae*, información procedente del Sistema Mundial de Vigilancia de la Resistencia a los Antimicrobianos de la OMS; de la red del Centro Europeo de Control de Enfermedades (ECDC) y de la base de datos de Liderazgo y Vigilancia de Pruebas Antimicrobianas de Pfizer. Utilizando el análisis de Bland-Altman, las pruebas *t* pareadas y las pruebas de los rangos con signo de Wilcoxon, se analizaron los datos sobre susceptibilidad y el número de concordancias aisladas entre plataformas.

**Resultados:**

De los 71 países que envían datos de manera activa a la OMS, 28 también los remiten a la base de datos de Pfizer; 19 al ECDC; y 16 a las tres plataformas. Los límites de concordancia entre las plataformas de la OMS y de Pfizer acerca de los datos sobre susceptibilidad organismo-país oscilaban entre el -26% y el 35%. Mientras que las susceptibilidades promedio entre las plataformas de la OMS y del ECDC no variaban (sesgo: 0%, intervalo de confianza del 95%: -2 a 2), la concordancia entre la susceptibilidad organismo-país era baja (límites de concordancia de -18 a 18%). Existen diferencias significativas en cifras aisladas notificadas por las plataformas de la OMS-Pfizer (media de la diferencia: 674, valor de* p*: < 0,001 y por las plataformas de la OMS–ECDC (media de la diferencia: 192, valor de* p*: 0,04).

**Conclusión:**

La notable heterogeneidad de los datos enviados a nivel nacional a las plataformas de vigilancia de la resistencia antimicrobiana utilizadas comúnmente compromete su validez, menoscabándose así las estrategias locales y mundiales sobre la resistencia antimicrobiana. Por tanto, se debe comprender y abordar la variabilidad de las plataformas de vigilancia, así como sus mecanismos subyacentes.

## ملخص

**الغرض:**

تقييم مدى اختلاف البيانات الوطنية للحساسية لمضادات الميكروبات المستخدمة لتوعية خطط العمل الوطنية عبر منصات المراقبة.

**الطريقة:**

قمنا بتحديد منصات المراقبة التفاعلية المتاحة ذات الوصول المفتوح وعبر الوطنية، ودققنا بياناتها وفقًا لضمان جودة البيانات الخاص بمنظمة الصحة العالمية (WHO): الوحدة رقم 1. قمنا بمقارنة قابلية استخدام المنصة واكتمال البيانات المتطابقة مع الوقت عن حساسية مضادات الميكروبات بالنسبة لأربعة أنواع من عزل الدم: *Escherichia coli *، و *Klebsiella pneumoniae *، و *Staphylococcus aureus * و *Streptococcus pneumoniae * من النظام العالمي التابع لمنظمة الصحة العالمية لمقاومة مضادات الميكروبات ومراقبة استخدامها، وشبكة المركز الأوروبي لمكافحة الأمراض (ECDC)، وقاعدة بيانات فايزر لقيادة ومراقبة اختبارات مضادات الميكروبات. باستخدام تحليل Bland-Altman واختبارات *t * المزدوجة، واختبارات تصنيف Wilcoxon الموقّع، قمنا بتقييم بيانات الحساسية، وعدد التوافقات العزل بين المنصات.

**النتائج:**

من بين 71 دولة تقدم البيانات بنشاط لمنظمة الصحة العالمية، تقدم 28 دولة منها أيضًا بيانات لقاعدة بيانات فايزر؛ و19 دولة تقدم البيانات لشبكة المركز الأوروبي لمكافحة الأمراض؛ وتقدم 16 دولة البيانات لجميع المنصات الثلاث. إن حدود التوافق بين بيانات منصتي منظمة الصحة العالمية وفايزر بشأن الحساسية المتعلقة بالكائنات الحية والدولة، تراوحت من %26 إلى %35. بينما لم تختلف الحساسيات المتوسطة لمنصتي منظمة الصحة العالمية، وشبكة المركز الأوروبي لمكافحة الأمراض (التحيز: %0، بفاصل الثقة مقداره 95: -2 إلى 2) كان التوافق بين حساسية الكائنات الحية والدولة منخفضًا (حدود التوافق −18 إلى %18). توجد فروق ذات دلالة إحصائية في أعداد العزل المبلغ عنها بين منظمة الصحة العالمية وفايزر (متوسط الاختلاف: 674، قيمة نسبة الاحتمال: أقل من 0.001 ومنصتي منظمة الصحة العالمية وشبكة المركز الأوروبي لمكافحة الأمراض (متوسط الاختلاف: 192، قيمة نسبة الاحتمال: 0.04).

**الاستنتاج:**

إن عدم التجانس الهائل في البيانات الوطنية المقدمة لمنصات مراقبة مقاومة مضادات الميكروبات شائعة الاستخدام يضعف صلاحيتها، وبالتالي يقوض استراتيجيات مقاومة مضادات الميكروبات المحلية والعالمية. وبالتالي، فنحن بحاجة إلى فهم تنوع منصة المراقبة، والآليات الكامنة وراءها، والتعامل مع ذلك كله.

## 摘要

**目的:**

旨在评估在不同监测平台上用于指导国家行动计划的国家抗微生物药物敏感性数据的差异。

**方法:**

根据世界卫生组织 (WHO) 数据质量保证：模块 1，我们确定了可用的开放获取、超国家的、交互式监测平台，并对其数据进行了交叉检查。我们比较了关于四种血液培养分离株的抗微生物药物敏感性的平台可用性和相同时间内的数据完整性，这四种分离株是：世卫组织全球抗微生物药物耐药性和使用监测系统、欧洲疾病预防控制中心 (ECDC) 网络和辉瑞抗微生物药物检测管理和监测数据库的*大肠杆菌、肺炎克雷伯菌、金黄色葡萄球菌和肺炎链球菌*。使用 Bland-Altman 分析、配对 t-检验和威尔科克森符号秩检验，我们评估了不同平台之间的药物敏感性数据和分离株一致性的数量。

**结果:**

在 71 个积极向世卫组织提交数据的国家中，有 28 个国家也向辉瑞的数据库提交了数据；19 个国家也向欧洲疾病预防控制中心提交了数据； 16 个国家向三个平台均提交了数据。世卫组织和辉瑞公司的国家生物药敏性数据平台之间的一致性差异范围为 -26% 至 35%。虽然世卫组织和欧洲疾病预防控制中心平台的平均敏感性没有差异（偏倚：0%；95% 置信区间：-2 至 2），但国家生物药物敏感性之间的一致性很低（一致性范围为 -18 至 18%）。世卫组织和辉瑞公司报告的分离株数量之间存在显著差异（差异均值：674, *P*-值：< 0.001，以及世卫组织和欧洲疾病预防控制中心平台之间存在的差异（差异均值：192, *P*-值：0.04）。

**结论:**

各国向常用的抗微生物药物耐药性监测平台提交的数据存在相当大的异质性，影响了它们的有效性，从而对当地和全球抗微生物药物耐药性战略的制定产生了不利影响。因此，我们需要了解并解决监测平台的变化性及其潜在机制。

## Резюме

**Цель:**

Оценить различия в национальных данных о восприимчивости к противомикробным препаратам, используемых для составления национальных планов действий, в зависимости от платформы эпиднадзора.

**Методы:**

Были определены доступные надгосударственные интерактивные платформы эпиднадзора с открытым доступом, а также проведена перекрестная проверка их данных в соответствии с «Обеспечением качества данных» Всемирной организации здравоохранения (ВОЗ): модуль 1. Сравнивались удобство использования платформы и полнота сопоставленных по времени данных о чувствительности к противомикробным препаратам четырех видов, выделенных из крови: *Escherichia coli, Klebsiella pneumoniae*, *Staphylococcus aureus* и Streptococcus pneumonia*e* из Глобальной системы надзора за устойчивостью к противомикробным препаратам и их использованием ВОЗ, сети Европейского центра по контролю заболеваний (ECDC) и базы данных Pfizer – Antimicrobial Testing Leadership and Surveillance. Для оценки данных о восприимчивости и количества совпадений изолятов между платформами использовались: анализ Бланда-Альтмана, парные *t*-критерии и ранговые критерии Уилкоксона.

**Результаты:**

Кроме того, данные из 71 страны, активно представляющей данные в ВОЗ, 28 стран также предоставляют данные в базу данных Pfizer, 19 – в ECDC, 16 – во все три платформы. Пределы согласия между платформами ВОЗ и Pfizer для данных о восприимчивости организмов по странам варьировались от –26 до 35%. Хотя средние значения восприимчивости платформ ВОЗ и ECDC не отличались (смещение: 0%, 95%-й ДИ: от –2 до 2), согласованность между восприимчивостью организма и страны была низкой (пределы согласования от –18 до 18%). Существуют значительные различия в количестве изолятов, зарегистрированных по данным ВОЗ-Pfizer (среднее значение разницы: 674, *P*-значение: < 0,001), и платформ ВОЗ-ECDC (среднее значение разницы: 192, *P*-значение: 0,04).

**Вывод:**

Значительная неоднородность данных, представляемых странами в рамках общепринятых платформ эпиднадзора за устойчивостью к противомикробным препаратам, ставит под сомнение их достоверность, тем самым препятствуя реализации местных и глобальных стратегий борьбы с устойчивостью к противомикробным препаратам. Следовательно, возникает необходимость в понимании и решении проблемы изменчивости платформы наблюдения и лежащих в ее основе механизмов.

## Introduction

Antimicrobial resistance is a growing threat to global public health.^1^ Recognizing the need for coordinated, evidence-based action, the 2015 World Health Assembly endorsed the *Global action plan on antimicrobial resistance*,^2^ with Member States agreeing to mandate the development and implementation of national action plans on antimicrobial resistance aligning human, animal and agricultural measures. 

Timely, accurate, relevant data are fundamental to informing country measures addressing antimicrobial resistance, hence the second of the five key global action plan implementation objectives is to “strengthen the knowledge and evidence base through surveillance and research.”^2^ Acknowledging that different countries may be at various starting points, the World Health Organization (WHO) has subsequently helped countries establish antimicrobial resistance surveillance and encouraged them to join their Global Antimicrobial Resistance and Use Surveillance System (known as GLASS).^3^ WHO also offers technical support, guidance, laboratory reporting standards and coordinating mechanisms for antimicrobial stewardship to countries needing strengthening of their diagnostic laboratory capacity. An aim of the support is to enable countries to submit clinically linked, nationally gathered data to WHO’s surveillance system, to describe both current and emerging resistance, and to monitor antimicrobial resistance and national action plans interventions.^4^ Initial assessment of developments of national surveillance capability following the release of the global action plan suggested some improvements, including in access to funding, but highlighted ongoing challenges and limited reporting outputs,^5^^–^^7^ particularly in low- and middle-income countries.^8^

In 2020, researchers were able to identify 71 separate international antimicrobial resistance surveillance platforms, ranging from targeted single disease surveillance, such as for tuberculosis, to supranational regional activity mirroring the aims of WHO’s surveillance system. However, very few offered readily available open-access data.^9^ These platforms included commercial platforms such as Pfizer’s antimicrobial testing leadership and surveillance database, which provides user-friendly, open-access and interactive visualization of available data, and has recently announced a public–private collaboration with the Wellcome Trust to address antimicrobial resistance in sub-Saharan Africa.^10^

As the coronavirus disease 2019 (COVID-19) pandemic comes under control, antimicrobial resistance must return to the forefront of the global health agenda. The pandemic has led to deterioration of antimicrobial susceptibility reporting activities^11^^,^^12^ and many of the national action plans have now expired. Now is an important moment to identify the current issues in global progress so that we can optimize the effectiveness of future actions; thus we need to evaluate the current surveillance platforms. We therefore analysed and compared international open-access antimicrobial resistance surveillance systems, using the WHO data quality assurance framework, dimension 3, that is, external comparison and/or cross-checks with other data sources.^13^ This analysis included assessing the consistency of the platforms’ data output of key pathogens.

## Methods

We conducted a search to identify potential, supranational, open-access, antimicrobial resistance interactive platforms for comparison with WHO’s global antimicrobial resistance and use surveillance system 2019 data (latest available year of reporting at the time of the search). The search was initially conducted in October 2021 and repeated in July 2022. First, we screened the 71 international antimicrobial resistance surveillance platforms identified in a 2020 review^9^ for suitability. We then searched the individual Member States’ health ministry (or equivalent) websites for involvement in additional supranational schemes. We screened the individual national action plans that were available in the WHO library of antimicrobial resistance national action plans^14^ for mentions of additional specific platforms. Finally, we conducted a general internet search using the Google search engine and the search words “AMR”, “antimicrobial resistance”, “national action plan”, “NAP” and the specific country of interest.

We used the following inclusion criteria: the platform had to (i) be entirely open access, interactive and web-based for reporting and visualizing antimicrobial resistance data; (ii) have data available to compare to those of 2019; (iii) represent at least supranational reporting of regional data; and (iv) contain data on blood culture isolates. The exclusion criteria were: not having open-access data via a readily open-access interactive platform; having no data available on the study period; or only partial reporting of data (organism of interest but not suitable antimicrobial).

### Analysis of surveillance data

For comparisons, the WHO data quality assurance framework suggests selecting a core set of four to five tracer indicators to identify any data completeness and quality issues.^13^ Thus, to enable direct comparison with other databases, we searched the WHO global antimicrobial resistance and use surveillance system for resistance data on four key blood stream infection organisms represented across the platforms: *Escherichia coli, Klebsiella pneumoniae*, *Staphylococcus aureus* and *Streptococcus pneumoniae.* The 2021 *Global antimicrobial resistance and use surveillance system (GLASS) report* states that the data collected for each data call (the last was in 2020 for participating countries) are antimicrobial susceptibility rates for the previous calendar year.^15^ We extracted the data on the number of isolates submitted for each species, the antimicrobial susceptibility results, age and gender of patients, number of patients tested and the origin of infection for each isolate. We then categorized these according to the system’s parameters of (i) no data available; (ii) < 70% data reported; or (iii) 70%–100% data reported. We also extracted the reported antimicrobial susceptibilities for the available indicators of resistance. For *E. coli* and *K. pneumoniae*, we selected the third-generation cephalosporin ceftazidime (or when not available, ceftriaxone); for *S. aureus*, oxacillin (or when not available, cefoxitin); and for *S. pneumoniae*, penicillin (or when not available, oxacillin). We selected the alternative antimicrobial when the primary selection was not being reported, or less than 30% of isolates having sensitivity results were available for primary selection. Six of the authors extracted these data across each identified platform, a different author covered each WHO region, and one author cross-checked all the regions.

### Comparison of platforms

To compare the strengths and weaknesses of platforms identified, we used pre-defined criteria. These criteria consisted of a broad overview of a combination of WHO Data Quality Assurance framework dimensions (qualitative consideration of data completeness, timeliness and internal consistency)^13^ and features specific to platform use, such as data accessibility and extraction, data representation and platform usability. We also pooled and summarized the qualitative comments from the data extractors to identify any strengths and weaknesses in visualization of data between platforms. Finally, we created a minimum recommended data set template as a potential method for increasing antimicrobial resistance reporting, engagement and representation.

### Statistical analysis

We conducted the statistical analysis and data visualizations in R version 4. 1.1 (R Foundation, Vienna, Austria), using the tidyverse, gtsummary, sf and rnaturalearth packages. We summarized the categorical variables as frequencies and percentages, and the continuous variables as medians and interquartile ranges (IQRs). We also stratified the countries’ key variables by WHO region.

We used Bland–Altman analysis to assess concordances between the proportion of isolate susceptibility that each country reported to WHO’s and identified platforms. We matched each organism with each country (hereafter referred to as organism–country combinations). This technique quantifies the concordances between two continuous measurements by calculating the mean difference (bias) and constructing limits of agreement (within which lie 95% of the differences between measurements).^16^ We then used paired *t*-tests to assess whether each country reported different mean susceptibility percentages for each organism to the two platforms. The number of isolates that each country reported to different platforms was summarized using medians and the median of the differences. We then compared these using Wilcoxon signed-rank tests to account for the paired data.

## Results

### Identification of platforms

We did not identify any additional platforms other than the 71 previous identified platforms.

In addition to WHO’s surveillance system, Pfizer’s antimicrobial testing leadership and surveillance database met the inclusion criteria and had a global scope. The European Centre for Disease Prevention and Control’s (ECDC’s) European antimicrobial resistance surveillance network was the only regional platform that met the inclusion criteria. Both WHO’s and Pfizer’s platforms enable the analysis of blood stream infection isolates independently of other specimen types, making possible direct comparison of the reported susceptibility rates for 2019 across countries. The ECDC network combines data on blood stream infections and cerebrospinal fluid. As the ECDC network feeds directly into WHO’s system, the aim of the comparison was to assess whether combining reported susceptibility estimates of important blood stream isolates and cerebrospinal fluid together resulted in any significant variance in reported organism susceptibility between the two platforms.

### Surveillance platform activity

**Fig. 1:**
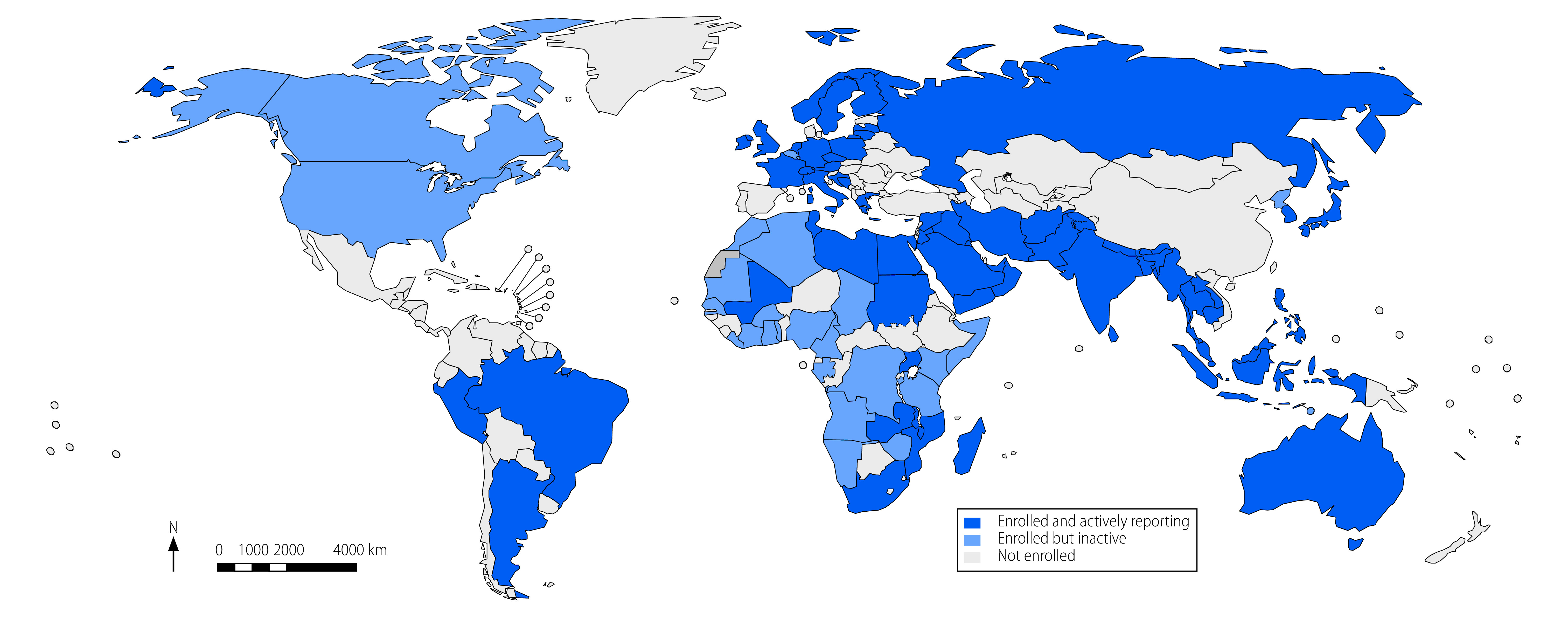
Reporting activity to global antimicrobial resistance and use surveillance system, August 2022

Note: We obtained evidence that 71 countries submitted surveillance data during the global antimicrobial resistance and use surveillance system’s 2020 data call. Countries that are enrolled in the system but have no data for the 2020 data call are also highlighted on the map.

As of August 2022, a total of 103 of the 194 (53.1%) WHO Member States have enrolled in WHO’s surveillance system. Of these, 100 (97.1%) have signed up to submit antimicrobial resistance surveillance data, and 18 (17.5%) have signed up to submit antimicrobial consumption data (Fig. 1). Of the 100 countries that committed to submit antimicrobial resistance surveillance data, 67 (67.0%) do so, with a further one country submitting partial data (1.0%). Three countries that have not enrolled also submit data (70/194; 36.1%; Fig. 1). Of the 71 countries actively submitting data to WHO’s surveillance system, 28 (39.4%) also submit to Pfizer’s platform and 19 (26.8%) submit to ECDC. Sixteen countries (22.5%) submit to all three platforms. 

### Surveillance data quality

Countries reporting on the four pre-set organisms and their associated antimicrobial sensitivity are presented in Table 1 (available at https://www.who.int/publications/journals/bulletin/). 

**Table 1:** Reported species susceptibility to open-access antimicrobial resistance surveillance platforms, by country, 2019

WHO region, country, organism	WHO’s surveillance system^a^				Pfizer’s surveillance database^b^				ECDC’s surveillance network^c^		
	Antibiotic	No. of isolates	Susceptibility, %		Antibiotic	No. of isolates	Susceptibility, %		Antibiotic	No. of isolates	Susceptibility, %
**African Region**											
South Africa											
*E. coli*	Ceftazidime	4 306	70.2		Ceftazidime	39	87.2		NR	NR	NR
*K. pneumoniae*	Ceftazidime	653	26.9		Ceftazidime	47	51.1		NR	NR	NR
*S. aureus*	Oxacillin	744	78.6		Oxacillin	46	89.1		NR	NR	NR
*S. pneumoniae*	Penicillin	6 315	72.2		Penicillin	9	77.8		NR	NR	NR
**Region of the Americas**											
Argentina											
*E. coli*	Ceftazidime	154	81.8		Ceftazidime	27	63.0		NR	NR	NR
*K. pneumoniae*	Ceftazidime	2 017	44.3		Ceftazidime	26	30.8		NR	NR	NR
*S. aureus*	Oxacillin	296	58.0		Oxacillin	47	57.5		NR	NR	NR
*S. pneumoniae*	Penicillin	1 732	75.4		Penicillin	2	100.0		NR	NR	NR
Brazil											
*E. coli*	Ceftazidime	214	88.2		Ceftazidime	39	76.9		NR	NR	NR
*K. pneumoniae*	Ceftazidime	166	39.8		Ceftazidime	53	35.9		NR	NR	NR
*S. aureus*	Oxacillin	6	79.4		Oxacillin	78	57.7		NR	NR	NR
*S. pneumoniae*	ND	6	ND		Penicillin	16	68.8		NR	NR	NR
**South-East Asia Region**											
India											
*E. coli*	Ceftazidime	ND	28.6		Ceftazidime	69	31.9		NR	NR	NR
*K. pneumoniae*	Ceftazidime	ND	40.0		Ceftazidime	57	29.8		NR	NR	NR
*S. aureus*	Cefoxitin	ND	44.2		Oxacillin	64	56.6		NR	NR	NR
*S. pneumoniae*	ND	ND	ND		Penicillin	17	29.4		NR	NR	NR
Thailand									NR	NR	NR
*E. coli*	Ceftazidime	1 121	71.7		Ceftazidime	47	72.3		NR	NR	NR
*K. pneumoniae*	Ceftazidime	2 453	61.2		Ceftazidime	39	53.9		NR	NR	NR
*S. aureus*	Cefoxitin	702	87.6		Oxacillin	37	73.0		NR	NR	NR
*S. pneumoniae*	Penicillin	180	61.4		Penicillin	2	0		NR	NR	NR
**European Region**											
Austria											
*E. coli*	Ceftazidime	2 382	90.4		NR	NR	NR		Cephalosporin^d^	61 06	90.3
*K. pneumoniae*	Ceftazidime	478	87.3		NR	NR	NR		Cephalosporin^d^	1 326	88.5
*S. aureus*	Oxacillin	478	94.7		NR	NR	NR		Meticillin	3 323	94.4
*S. pneumoniae*	Penicillin	1 305	93.5		NR	NR	NR		Penicillin	458	93.2
Croatia											
*E. coli*	Ceftazidime	143	84.1		Ceftazidime	63	84.1		Cephalosporin^d^	1 085	83.0
*K. pneumoniae*	Ceftazidime	1 111	48.1		Ceftazidime	52	40.4		Cephalosporin^d^	317	45.4
*S. aureus*	Cefoxitin	153	75.1		Oxacillin	90	78.9		Meticillin	358	75.1
*S. pneumoniae*	Penicillin	358	72.8		Penicillin	14	92.7		Penicillin	154	79.9
Cyprus											
*E. coli*	Ceftazidime	60	83.5		NR	NR	NR		Cephalosporin^d^	92	79.3
*K. pneumoniae*	Ceftazidime	8	54.6		NR	NR	NR		Cephalosporin^d^	60	50.0
*S. aureus*	Oxacillin	32	0		NR	NR	NR		Meticillin	58	63.8
*S. pneumoniae*	ND	92	ND		NR	NR	NR		Penicillin	2	0
Czechia											
*E. coli*	Ceftazidime	95	84.0		Ceftazidime	33	84.9		Cephalosporin^d^	3 557	82.7
*K. pneumoniae*	Ceftazidime	387	84.1		Ceftazidime	24	54.2		Cephalosporin^d^	1 563	47.9
*S. aureus*	Oxacillin	387	88.0		Oxacillin	38	89.5		Meticillin	2 108	87.4
*S. pneumoniae*	Penicillin	1 563	94.9		Penicillin	9	77.8		Penicillin	387	95.1
Finland											
*E. coli*	Ceftazidime	1 494	92.3		NR	NR	NR		Cephalosporin^d^	5 413	91.3
*K. pneumoniae*	Ceftazidime	628	92.4		NR	NR	NR		Cephalosporin^d^	868	91.8
*S. aureus*	Oxacillin	957	97.7		NR	NR	NR		Meticillin	53	97.9
*S. pneumoniae*	Penicillin	6 225	88.1		NR	NR	NR		Penicillin	594	88.0
France											
*E. coli*	Ceftazidime	1 264	91.3		Ceftazidime	110	94.6		Cephalosporin^d^	13 019	90.2
*K. pneumoniae*	Ceftazidime	13 097	69.1		Ceftazidime	82	72.0		Cephalosporin^d^	3 075	68.1
*S. aureus*	Oxacillin	1 264	88.4		Oxacillin	140	88.6		Meticillin	6 467	88.4
*S. pneumoniae*	Penicillin	472	74.7		Penicillin	71	77.5		Penicillin	1 264	74.7
Germany											
*E. coli*	Ceftazidime	1 981	88.2		Ceftazidime	27	96.3		Cephalosporin^d^	23 413	87.9
*K. pneumoniae*	Ceftazidime	10 939	86.7		Ceftazidime	25	76.0		Cephalosporin^d^	4 719	86.5
*S. aureus*	Oxacillin	23 387	93.2		Oxacillin	27	88.9		Meticillin	11 950	93.3
*S. pneumoniae*	Penicillin	154	94.3		Penicillin	20	95.0		Penicillin	1 962	94.3
Greece											
*E. coli*	Ceftazidime	1 946	83.8		Ceftazidime	15	100.0		Cephalosporin^d^	190	80.0
*K. pneumoniae*	Ceftazidime	1 588	35.4		Ceftazidime	26	11.5		Cephalosporin^d^	310	32.6
*S. aureus*	Oxacillin	1 059	56.6		Oxacillin	26	76.9		Meticillin	170	37.6
*S. pneumoniae*	ND	1221	ND		Penicillin	9	88.9		Penicillin	0	0
Ireland											
*E. coli*	Ceftazidime	885	85.9		Ceftazidime	8	87.5		Cephalosporin^d^	3 231	86.1
*K. pneumoniae*	Ceftazidime	348	81.8		Ceftazidime	13	46.2		Cephalosporin^d^	527	80.6
*S. aureus*	Oxacillin	64	87.2		Oxacillin	13	92.3		Meticillin	1 146	87.4
*S. pneumoniae*	Penicillin	3 229	85.6		Penicillin	4	100.0		Penicillin	348	85.6
Italy											
*E. coli*	Ceftazidime	8 356	70.3		Ceftazidime	74	75.7		Cephalosporin^d^	18 409	68.2
*K. pneumoniae*	Ceftazidime	1 639	42.3		Ceftazidime	73	30.1		Cephalosporin^d^	7 699	40.8
*S. aureus*	Oxacillin	1 166	64.8		Oxacillin	119	63.9		Meticillin	9 681	65.7
*S. pneumoniae*	Penicillin	18 404	88.1		Penicillin	38	81.6		Penicillin	1 017	88.1
Latvia											
*E. coli*	Ceftazidime	640	81.6		Ceftazidime	9	66.7		Cephalosporin^d^	442	79.9
*K. pneumoniae*	Ceftazidime	604	62.9		Ceftazidime	9	77.8		Cephalosporin^d^	198	63.1
*S. aureus*	Cefoxitin	112	92.0		Oxacillin	14	100.0		Meticillin	421	92.6
*S. pneumoniae*	Penicillin	112	88.0		Penicillin	10	90.0		Penicillin	79	89.9
Lithuania											
*E. coli*	Ceftazidime	439	86.9		Ceftazidime	27	77.8		Cephalosporin^d^	1 132	84.5
*K. pneumoniae*	Ceftazidime	120	45.0		Ceftazidime	22	50.0		Cephalosporin^d^	440	43.2
*S. aureus*	Cefoxitin	107	90.7		Oxacillin	52	88.5		Meticillin	656	90.7
*S. pneumoniae*	Penicillin	120	89.2		Penicillin	13	84.6		Penicillin	120	89.2
Luxembourg											
*E. coli*	Ceftazidime	38	88.0		NR	NR	NR		Cephalosporin^d^	1 132	84.5
*K. pneumoniae*	Ceftazidime	209	73.8		NR	NR	NR		Cephalosporin^d^	103	73.8
*S. aureus*	Oxacillin	38	93.8		NR	NR	NR		Meticillin	209	93.8
*S. pneumoniae*	Penicillin	10	79.0		NR	NR	NR		Penicillin	38	78.9
Malta											
*E. coli*	Ceftazidime	9	81.3		NR	NR	NR		Cephalosporin^d^	332	82.2
*K. pneumoniae*	Ceftazidime	358	57.7		NR	NR	NR		Cephalosporin^d^	129	58.9
*S. aureus*	Oxacillin	16	76.6		NR	NR	NR		Meticillin	75	76.0
*S. pneumoniae*	Penicillin	77	63.0		NR	NR	NR		Penicillin	27	66.7
Netherlands (Kingdom of the)											
*E. coli*	Ceftazidime	7 300	92.6		Ceftazidime	18	100.0		Cephalosporin^d^	7 300	92.0
*K. pneumoniae*	Ceftazidime	1 434	90.2		Ceftazidime	8	87.5		Cephalosporin^d^	1 434	89.5
*S. aureus*	Oxacillin	1 256	98.4		Oxacillin	18	100.0		Meticillin	3 221	98.4
*S. pneumoniae*	Penicillin	2 627	96.1		Penicillin	25	100.0		Penicillin	1 360	96.0
Norway											
*E. coli*	Ceftazidime	1 106	93.9		NR	NR	NR		Cephalosporin^d^	4 075	93.2
*K. pneumoniae*	Ceftazidime	62	91.3		NR	NR	NR		Cephalosporin^d^	832	91.0
*S. aureus*	Oxacillin	504	99.0		NR	NR	NR		Meticillin	1 644	98.9
*S. pneumoniae*	Penicillin	23	93.7		NR	NR	NR		Penicillin	504	93.7
Poland											
*E. coli*	Ceftazidime	65	83.1		Ceftazidime	20	95.0		Cephalosporin^d^	2 803	82.2
*K. pneumoniae*	Ceftazidime	1 161	41.5		Ceftazidime	25	24.0		Cephalosporin^d^	1 166	40.8
*S. aureus*	Cefoxitin	254	85.1		Oxacillin	43	86.1		Meticillin	1 841	85.1
*S. pneumoniae*	Penicillin	319	85.3		Penicillin	21	76.2		Penicillin	310	84.5
Russian Federation											
*E. coli*	Ceftazidime	216	53.3		Ceftazidime	41	24.4		NR	NR	NR
*K. pneumoniae*	Ceftazidime	5	20.5		Ceftazidime	60	23.3		NR	NR	NR
*S. aureus*	Cefoxitin	23	76.7		Oxacillin	95	74.7		NR	NR	NR
*S. pneumoniae*	Penicillin	418	93.3		Penicillin	7	85.7		NR	NR	NR
Sweden											
*E. coli*	Ceftazidime	1 069	92.3		Ceftazidime	ND	ND		Cephalosporin^d^	9 419	91.9
*K. pneumoniae*	Ceftazidime	5 948	91.1		Ceftazidime	13	92.3		Cephalosporin^d^	1 795	90.6
*S. aureus*	Cefoxitin	9 421	98.2		Oxacillin	ND	ND		Meticillin	5 948	98.8
*S. pneumoniae*	Penicillin	253	93.5		Penicillin	2	50.0		Penicillin	1 070	93.5
Switzerland											
*E. coli*	Ceftazidime	63	89.7		Ceftazidime	24	83.3		NR	NR	NR
*K. pneumoniae*	Ceftazidime	75	91.3		Ceftazidime	11	81.8		NR	NR	NR
*S. aureus*	Cefoxitin	6 048	96.5		Oxacillin	10	90.0		NR	NR	NR
*S. pneumoniae*	Penicillin	726	94.8		Penicillin	9	88.9		NR	NR	NR
United Kingdom											
*E. coli*	Ceftazidime	1 932	87.5		Ceftazidime	56	94.6		Cephalosporin^d^	26 593	87.4
*K. pneumoniae*	Ceftazidime	705	85.3		Ceftazidime	36	77.8		Cephalosporin^d^	4 867	85.4
*S. aureus*	Cefoxitin	3 556	89.6		Oxacillin	40	92.5		Meticillin	9 114	94.0
*S. pneumoniae*	Penicillin	5 085	94.7		Penicillin	16	93.8		Penicillin	3 667	94.5
**Eastern Mediterranean Region**											
Jordan											
*E. coli*	Ceftriaxone	183	33.6		Ceftriaxone	ND	ND		NR	NR	NR
*K. pneumoniae*	Ceftriaxone	195	26.0		Ceftriaxone	ND	ND		NR	NR	NR
*S. aureus*	Oxacillin	137	27.6		Oxacillin	ND	ND		NR	NR	NR
*S. pneumoniae*	Ceftriaxone	97	90.0		Penicillin	ND	ND		NR	NR	NR
Qatar											
*E. coli*	Ceftazidime	ND	62.2		Ceftazidime	18	22.2		NR	NR	NR
*K. pneumoniae*	Ceftazidime	ND	71.7		Ceftazidime	11	54.6		NR	NR	NR
*S. aureus*	Oxacillin	ND	66.2		Oxacillin	29	51.7		NR	NR	NR
*S. pneumoniae*	Penicillin	ND	79.0		Penicillin	17	64.7		NR	NR	NR
Saudi Arabia											
*E. coli*	Ceftazidime	591	42.1		Ceftazidime	6	50.0		NR	NR	NR
*K. pneumoniae*	Ceftazidime	42	27.8		Ceftazidime	8	37.5		NR	NR	NR
*S. aureus*	Cefoxitin	60	51.1		Oxacillin	6	50.0		NR	NR	NR
*S. pneumoniae*	Oxacillin	307	57.9		Penicillin	1	0		NR	NR	NR
**Western Pacific Region**											
Australia											
*E. coli*	Ceftazidime	3 157	87.0		Ceftazidime	24	79.2		NR	NR	NR
*K. pneumoniae*	Ceftazidime	4 914	90.1		Ceftazidime	18	94.4		NR	NR	NR
*S. aureus*	Cefoxitin	1 143	81.5		Oxacillin	17	100.0		NR	NR	NR
*S. pneumoniae*	ND	110	ND		Penicillin	32	96.9		NR	NR	NR
Japan											
*E. coli*	Ceftazidime	26 176	86.3		Ceftazidime	21	61.9		NR	NR	NR
*K. pneumoniae*	Ceftazidime	78 923	95.5		Ceftazidime	14	92.9		NR	NR	NR
*S. aureus*	Oxacillin	608	63.6		Oxacillin	34	64.7		NR	NR	NR
*S. pneumoniae*	Penicillin	3 241	98.7		Penicillin	4	100.0		NR	NR	NR
Malaysia											
*E. coli*	Ceftazidime	699	75.7		Ceftazidime	20	60.0		NR	NR	NR
*K. pneumoniae*	Ceftazidime	8 875	66.3		Ceftazidime	22	54.6		NR	NR	NR
*S. aureus*	Oxacillin	2 001	81.7		Oxacillin	28	57.1		NR	NR	NR
*S. pneumoniae*	Penicillin	1 079	86.3		Penicillin	11	100.0		NR	NR	NR
Philippines											
*E. coli*	Ceftazidime	256	66.2		Ceftazidime	20	60.0		NR	NR	NR
*K. pneumoniae*	Ceftazidime	1 583	46.2		Ceftazidime	12	58.3		NR	NR	NR
*S. aureus*	Oxacillin	166	49.1		Oxacillin	37	56.8		NR	NR	NR
*S. pneumoniae*	Penicillin	1 420	86.2		Penicillin	6	83.3		NR	NR	NR
Republic of Korea											
*E. coli*	Ceftazidime	683	79.9		Ceftazidime	54	63.0		NR	NR	NR
*K. pneumoniae*	Ceftazidime	716	80.7		Ceftazidime	6	66.7		NR	NR	NR
*S. aureus*	Cefoxitin	225	51.4		Oxacillin	27	51.9		NR	NR	NR
*S. pneumoniae*	Penicillin	47	58.3		Penicillin	3	66.7		NR	NR	NR

ECDC: European Centre for Disease Prevention and Control; NR: not reported; WHO: World Health Organization.

^a^ Full name: global antimicrobial resistance and use surveillance system.

^b^ Full name: antimicrobial testing leadership and surveillance.

^c^ Full name: European antimicrobial resistance surveillance network.

^d^ 3^rd^ generation cephalosporin.

Note: Cephalosporin antibiotic is 3

Examining the proportion of organism–country combinations that had 70%–100% data reported to WHO’s surveillance system, we found that: 96.8% (271) of combinations had antimicrobial sensitivity data; 88.9% (249) had information on gender; 83.6% (234) had information on age; 35.7% (100) had information on the total numbers of patients tested; and only 21.4% (60) had information on infection origin. The Western Pacific and African Regions provided data more consistently on the numbers of patients tested; the South-East Asia, European, Western Pacific Regions provided data on age, and the Western Pacific Region provided data on infection origin. Across the Regions of the Americas, the reliability of the available sensitivity and age data was comparatively low, whereas in the European Region, the reliability of the available infection origin data was notably low (available in the online repository).^17^ Across WHO regions, significant variation was noted in the susceptibility data regarding *E. coli*, *K. pneumoniae* and *S. aureus*, but less variation regarding the *S. pneumoniae* data (Table 2).

**Table 2:** Reported organism susceptibility data in WHO global antimicrobial resistance and use surveillance system across WHO regions, 2019

Organism	WHO region, median % (IQR)					
	African (8 countries)	Americas (4 countries)	Eastern Mediterranean (18 countries)	European (24 countries)	South-East Asian (8 countries)	Western Pacific (9 countries)
*Escherichia coli*	60 (55 to 72)	85 (83 to 87)	46 (34 to 54)	85 (82 to 90)	42 (28 to 47)	76 (66 to 83)
*Klebsiella pneumoniae*	22 (8 to 34)	42 (41 to 43)	30 (26 to 46)	66 (42 to 87)	35 (23 to 42)	77 (67 to 85)
*Staphylococcus aureus*	84 (78 to 96)	69 (63 to 74)	51 (33 to 62)	89 (77 to 94)	57 (48 to 70)	76 (61 to 83)
*Streptococcus pneumoniae*	72 (63 to 72)	75 (75 to 75)	84 (62 to 92)	88 (79 to 94)	80 (71 to 85)	86 (69 to 86)

IQR: interquartile range; WHO: World Health Organization.

Note: Median susceptibilities are presented as a percentage of susceptible isolates for each WHO region for *E. coli* and *K. pneumoniae* and third-generation cephalosporins, *S. aureus* and oxacillin, and *S. pneumoniae* and penicillin. Countries are reported in Table 1.

**Fig. 2:**
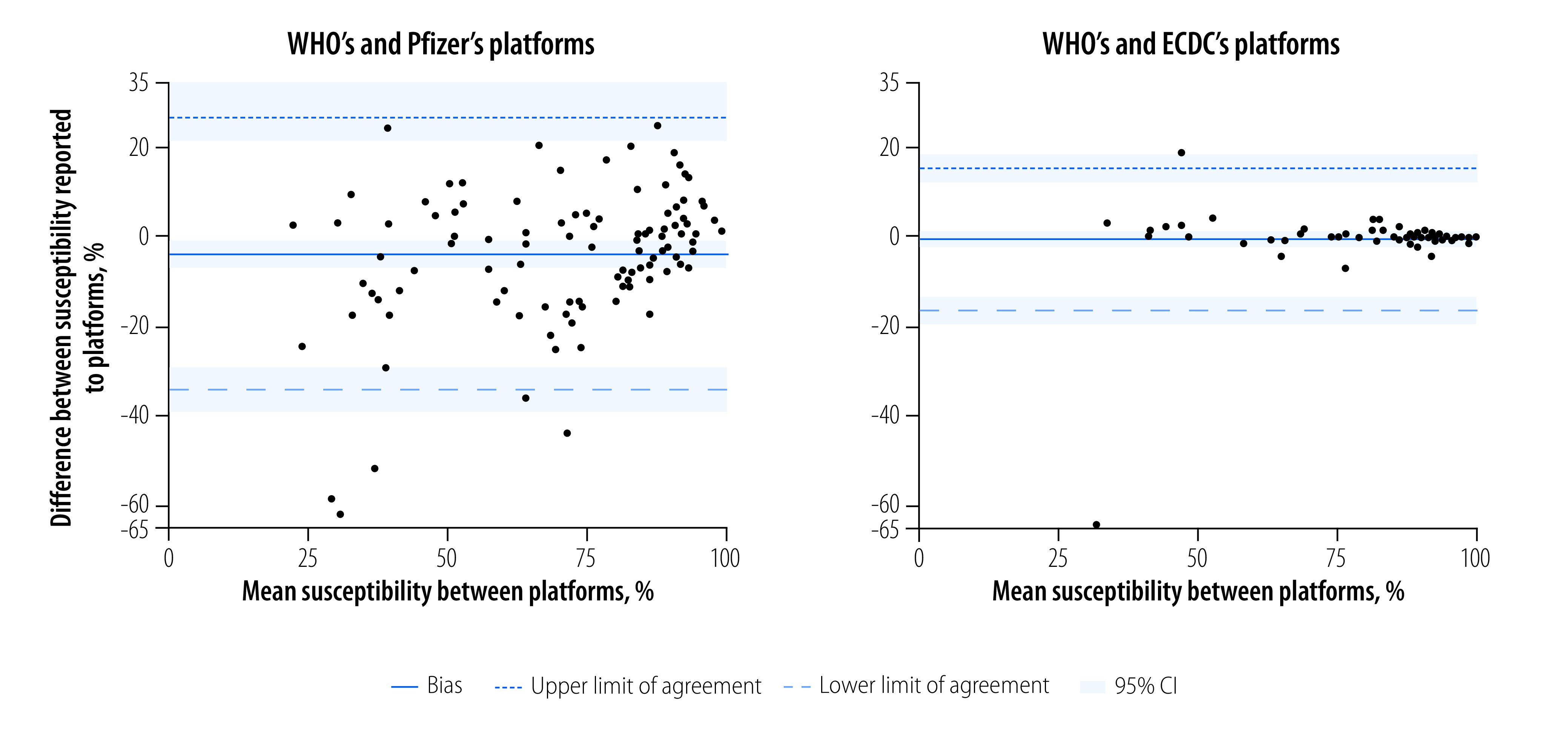
Bland–Altman plots demonstrating variation in organism–country susceptibility results between supranational open-access antimicrobial resistance platforms, 2019

CI: confidence interval; ECDC: European Centre for Disease Prevention and Control; WHO: World Health Organization.

Note: Included databases are WHO’s global antimicrobial resistance and use surveillance system, Pfizer’s antimicrobial testing leadership and surveillance and ECDC’s European antimicrobial resistance surveillance network. The y-axes show the differences between the susceptibilities of each organism–country combination result (i.e. the difference between the *E. coli* susceptibility to third-generation cephalosporins for Japan reported to WHO’s system, and those reported to Pfizer’s platform).

Comparison of the platform data showed that the data submitted to WHO’s surveillance system were more antimicrobial susceptible than average data submitted to Pfizer’s platform (bias: 4%, 95% confidence interval, CI: 1 to 7). The concordance between these two platforms’ organism–country susceptibilities was extremely low, with 95% limits of agreement ranging from −26% to 35%. This result indicates that for 95% of organism–country combinations, the absolute difference between the susceptibility reported to WHO’s surveillance system and that reported to Pfizer’s platform was possibly as great as 35% (Fig. 2). We found no evidence that WHO’s and ECDC’s surveillance platforms had different mean susceptibilities (bias: 0%; 95% CI: −2% to 2%). However, the concordance between the organism–country combinations was low, with 95% limits of agreement from −18% to 18%, even though two outlying data points primarily drove this result (Table 3).

**Table 3:** Comparison of median of differences in antimicrobial susceptibility and number of isolates reported to supranational open-access surveillance databases, 2019

Comparison, organism	Susceptibility %				No. of isolates		
	Median		Median of differences (IQR)*		Median		Median of differences (IQR)*
	WHO^a^	Comparator platform			WHO^a^	Comparator platform	
**WHO^a^ vs Pfizer^b^ (28 countries)**							
*Escherichia coli*	83.8	77.4	−0.3 (−6.7 to 14.0)		699	27	655.0 (175.0 to 1936.8)
*Klebsiella pneumoniae*	61.2	54.2	7.3 (−3.7 to 12.0)		705	24	1136.0 (363.0 to 2414.0)
*Staphylococcus aureus*	79.4	77.9	−0.7 (−4.8 to 2.2)		478	37	461.5 (89.2 to 1124.5)
*Streptococcus pneumoniae*	88.0	84.6	0.9 (−4.8 to 8.3)		472	10	411.0 (178.0 to 1730.0)
**WHO^a^ vs ECDC^c^ (19 countries)**							
*Escherichia coli*	86.9	86.1	1.0 (0.3 to 1.5)		1069	3557	−2738.0 (−6134.5 to −388.5)
*Klebsiella pneumoniae*	69.0	68.1	0.6 (0.1 to 1.4)		628	868	−5.0 (−545.0 to 600.0)
*Staphylococcus aureus*	89.6	90.7	0 (−0.4 to 0)		478	1644	−549.0 (−1843.0 to −42.5)
*Streptococcus pneumoniae*	88.1	88.1	0 (−0.1 to 0.1)		358	387	90.0 (−14.0 to 1244.0)

ECDC: European Centre for Disease Prevention and Control; IQR: interquartile range; WHO: World Health Organization.

^a^ Full name: global antimicrobial resistance and use surveillance system.

^b^ Full name: antimicrobial testing leadership and surveillance.

^c^ Full name: European antimicrobial resistance surveillance network.

Note: The raw data used to calculate the medians are available in Table 1. The values pertaining to WHO’s system may vary between the two comparisons because the same countries do not report to both Pfizer’s and ECDC’s platforms.

We found significant evidence that countries report different numbers of isolates to WHO’s surveillance system and Pfizer’s platform (*P*-value: < 0.001), and significant evidence that countries report different numbers of isolates to the WHO and ECDC platforms (*P*-value: 0.04). Comparison of the number of isolates reported to the WHO and Pfizer platforms revealed that the median of the differences was 674 isolates (IQR: 175 to 1917 isolates). Comparison of the number of isolates reported to the WHO and ECDC platforms revealed that the median of differences was 192 isolates (IQR: −273 to 1743 isolates). Table 3 presents a summary of statistics stratified by organism.

### Comparison of platforms 

Table 4 presents the overall aims of each platform, and their weaknesses and strengths regarding consistency in presentation and accessibility of data; reporting standards; completeness and quality of data; and consistency of data across key demographic indicators.

**Table 4:** Comparison of key usability features of open-access, international antimicrobial resistance surveillance platforms

Dimensions, perceived strength or weakness	WHO’s global antimicrobial resistance and use surveillance system	Antimicrobial testing leadership and surveillance database	European antimicrobial resistance surveillance platform
**Broad aims**	Global surveillance system using national-level routine surveillance data to estimate antimicrobial resistance burden and identify emerging resistance across sectors by using the One Health approach	Provides a privately funded service to assess emerging bacterial and fungal resistance through a user-friendly website and mobile application interface. Data are drawn from regions participating in three surveillance programmes^a^	Large, publicly funded continental surveillance platform that aims to collect comparable, representative, temporospatial data to timely identify antimicrobial resistance trends across Europe, inform policy and optimize national surveillance programmes
**Consistency in presentation and accessibility**			
Strength	Qualitative summary pages for each country provide detailed overview (i.e. no. of reporting rounds per year, no. of reporting stations) of available data	Representation of changes in antimicrobial resistance over time can be easily visualized using embedded interactive heat maps. Data extraction in multiple formats	Easy-to-use interface requiring minimal learning. Data visualization provided in multiple tabular and graphical formats on one interactive page to provide regional overview. Data presented using clearly defined antimicrobial resistance indicators for clinically important mechanisms
Weakness	Data retrieved for individual countries are displayed separately with limited visualization of trends or differences across more than one country; the platform is embedded within a webpage, meaning it can be more difficult to visualize complete data on one page	A period of learning time for end-users wishing to optimize data extraction across different formats was felt to be required when compared to other platforms	Limited ability to visualize all collated data for individual countries
**Antimicrobial susceptibility reporting standards**			
Strength	Antimicrobial susceptibility data provided according to Clinical and Laboratory Standards Institute and/or European Committee on Antimicrobial Susceptibility Testing interpretation rules, with confirmation of reporting standards used by each country in periodic reports	Users can switch between Clinical and Laboratory Standards Institute and European Committee on Antimicrobial Susceptibility Testing susceptibility cut-offs to allow greater flexibility in comparing country susceptibility results	Unified European Committee on Antimicrobial Susceptibility Testing reporting from 2019 onwards
Weakness	Potential for misinterpreting susceptibility data when comparing countries that report to both Clinical and Laboratory Standards Institute and European Committee on Antimicrobial Susceptibility Testing standards	None identified	Mixed Clinical and Laboratory Standards Institute and European Committee on Antimicrobial Susceptibility Testing reporting before 2019
**Completeness of antimicrobial susceptibility data**			
Strength	A detailed periodic report providing an overview of changes in data is provided. Option to search by a range of sample types, including blood, genital, urine and stool	Data reports can be prepared for detailed and discrete combinations of pathogens, specific antimicrobial susceptibilities, time periods and countries	Provides a detailed periodic report with an overview of changes in data. Data are presented using clearly defined indicator antimicrobial agents for clinically important antimicrobial resistance mechanisms, i.e. third-generation cephalosporins as a screening indicator for possible extended spectrum β lactamases
Weakness	Infection origin and overall no. of patients tested variably presented qualitatively only, or qualitatively and quantitatively. Difficult for users to interpret antimicrobial resistance results for different origins (community vs hospital) of infection, despite intent that such data are included in the surveillance reports^15^	Data on infection source are unavailable. Available antimicrobial susceptibility reporting can limit analysis of changes in indicator agents	Data on infection source are unavailable. Data presentation is restricted to pooled invasive cerebrospinal and blood isolates only
**Quality of antimicrobial susceptibility data**			
Strength	Indication of available susceptibility data for each antibiotic is provided with a cut-off of less or greater than 30%. If data set contains < 10 patients, no susceptibility value is provided	Data can be analysed for highly specific situations including pathogen–antimicrobial susceptibility combinations by age, source and location	Cut-offs are applied for minimum required pathogen-antimicrobial combination reporting to reduce misleading data representation
Weakness	Limited ability to view data across specific time periods	Susceptibility data may be presented for very small sample sets, risking misinterpretation of available data. Data volume for any given year is substantially less than the two other platforms, limiting interpretation. Data collection strategy through specific studies limits representation of data to national susceptibility rates	Data presented are not disaggregated by community or hospital source
**Consistency of data across key demographic indicators**			
Strength	Provides antimicrobial resistance-stratified frequency data (per 100 000 tested patients) for age and gender with CIs for a set of pathogen–antimicrobial combinations. Presents qualitative demographics, infection source and no. of patients tested for isolates. CIs provided for antimicrobial sensitivity testing data	Data search functions by hospital division (i.e. surgical, medical, intensive care, as well as non-hospital health-care environments such as nursing homes). Data search function by source of infection	Option to assess demographic data quality as discrete percentages (tabular) and via a graphical heat map with an upper range > 90% cut-off
Weakness	Demographics, no. of patients tested and infection origin data are limited by qualitative presentation, with a low upper-band cut-off of > 70% data availability. Limited ability to apply demographic data to susceptibility data	No gender data available. Available data limited to health-care environments	Limited ability to apply demographic data to pathogen–antimicrobial combinations

CI: confidence interval; WHO: World Health Organization.

^a^ Surveillance programmes that inform Pfizer’s platform include Tigecycline evaluation Surveillance Trial, Assessing Worldwide Antimicrobial Resistance Evaluation and International Network for Optimal Resistance Monitoring.^18^

Note: Perceived strengths and weaknesses of evaluated antimicrobial resistance surveillance platforms have been considered according to use for data extraction, broadly considering topics that reflect relatable elements of the WHO Data Quality Assurance Framework and general usability.

### Proposed data set requirements

As we found that the data representativeness and data quality vary across the platforms and WHO regions, we propose a minimum data set requirement for reporting blood stream infection antimicrobial resistance data in the form of a potential template (Table 5). This template focuses on reporting at least the four blood stream infection organisms analysed here alongside the key antimicrobial susceptibility indicator data and the baseline demographic data.

**Table 5:** Proposed minimum and optimal data requirement for antimicrobial resistance surveillance reporting for international systems/platforms

Data category	Proposed minimum data requirement – to ensure accuracy and consistency	Proposed optimum data set once effective surveillance platform established
Time interval	Annual	Annual
Pathogen–antimicrobial combinations	*Escherichia coli* and *Klebsiella pneumoniae* - third-generation cephalosporin (cefotaxime or cefpodoxime or ceftriaxone and ceftazidime) - carbapenem (imipenem and/or meropenem) - a quinolone (ciprofloxacin, levofloxacin and/or ofloxacin) - aminoglycoside (gentamicin or amikacin) *Staphylococcus aureus* - Methicillin-resistant *Staphylococcus aureus* indicator (oxacillin or cefoxitin) *Streptococcus pneumoniae* - Penicillin (Penicillin G or benzylpenicillin)	*Candida* species - Fluconazole *Enterococcus faecalis* and *faecium* - Vancomycin or teicolplanin *Pseudomonas aeruginosa* - Beta-lactam (ceftazidime and/or piperacillin-tazobactam and/or meropenem) *Acinetobacter baumannii* - Meropenem
Source of blood stream infection	Provide confirmation on whether source was identified (reported as yes or no).	Consider option for data matching pathogen results with source of infection (i.e. urinary, biliary, soft tissue skin infection).
Origin of infection	Provide data on hospital or community origin of infection	Consider option of splitting community data to include long-term care facilities. Disaggregate hospital data by specialty, e.g. infections arising from medical wards, surgical wards, rehabilitation wards and intensive care units
Demographics of interest	Gender and age (grouped)	Age by year. Standard ethnicity metric to capture variation in different populations across and within countries

Notes: A suggested approach to a minimum data set requirement for countries developing national surveillance capability, with antimicrobial indicators to provide both flexibility and comparability across countries. Minimum data set requirements could complement a periodic national survey approach and assist harmonization across platforms. A desirable data set is also postulated for countries with established platforms to further optimize surveillance.

## Discussion

Our findings suggest considerable inconsistencies between the surveillance data in supranational observatory platforms, raising concerns about their reliability for reflecting national or local community needs. In 2021, WHO announced a renewed *Call to action on antimicrobial resistance*, seeking to accelerate the commitments made previously to tackling this global public health concern, using the One Health approach but considering the varied circumstances of individual countries.^19^ Having garnered the active support of 113 Member States, an opportunity now exists to identify and address the deficiencies in antimicrobial resistance surveillance data.

Making flexible, open-access antimicrobial resistance surveillance platforms that require minimum entry available to reporting laboratories to facilitate accuracy, rather than striving for unachievable completeness in surveillance data submission, could enable countries lacking the diagnostic or workforce capacity to obtain meaningful surveillance data for national measures and international collaboration.^20^ The substantial discrepancies between surveillance platforms in species susceptibility within countries revealed here reduces the ability to reliably monitor any development in national, regional and global antimicrobial resistance patterns. This variability must be addressed without delay if we are to ensure reliability of private or public platform outputs and to avoid misdirecting antimicrobial stewardship and research on antimicrobial resistance and antimicrobial stewardship at the national and regional levels.^10^^,^^21^ The wide variation between countries in the amount of species data submitted to each platform highlights sample selection bias. In addition, smaller sample sizes are unlikely to represent any variability in inter-city or regional resistance.^22^^–^^24^

To improve the submission of reliable data, we suggest that laboratories should be provided with a minimum required reporting data set template that includes only key pathogens. This approach may be especially useful in invigorating surveillance activity in those countries whose capabilities are still in the early development stage. This template could also stipulate that only the susceptibility of indicator antimicrobials is required (as in the ECDC’s network), which would help countries focus on susceptibility testing strategies when funding is scarce but allow for regional variation in the selection of appropriate/available indicator antimicrobial agents. WHO has recently published methodological principles for nationally representative surveys of antimicrobial resistant blood stream infections,^25^ which may be further facilitated by a minimal data set approach. While improving diagnostic capability is likely to require substantial financial investment in some situations, this document provides timely guidance for countries with limited surveillance infrastructures to undertake periodic strategic sampling of defined population subsets to address reporting bias issues.^25^ This approach could be combined with restricting national data reporting requirements to a minimum and optimizing available funds to ensure adequate diagnostics to support this minimum data set. Subsequently, platforms should be adapted to include information on source data type (periodic survey versus routine national data) and should streamline upload mechanisms for minimum versus expanded data sets. Sharing the lessons learned with regional partners and considering the adoption of a periodic survey method potentially coordinated by the regional WHO offices will be integral for maximizing efforts and avoiding duplication of work.

Although capacity strengthening is essential for developing surveillance platforms, giving a clinical context to the available data could also be a priority for established platforms.^5^ A major benefit of WHO’s surveillance system is the option to submit isolate-level clinical information, and although demographic data are often available, information on infection origin (particularly in Europe) and the total number of isolates tested is often lacking. Combining clinical information and antimicrobial resistance data can improve the scope and applicability of individualized antimicrobial stewardship guidelines.^20^ Even accounting for the additional time and resource burden associated with submitting data to WHO’s surveillance system in a tertiary hospital in Thailand, for example, the authors consider WHO’s system outputs superior in contributing to antimicrobial guideline development.^20^ Accurate interpretation of the variation in bacteraemia isolation rates during COVID-19 has been complicated by imprecise denominator estimates, even in countries that are able to provide the most comprehensive data, and this highlights the importance of improving data quality across the board.^26^ Multiple platform use is likely to further challenge the already limited workforce capacity, and if opportunities to optimize data quality are not taken, alternative platforms could seek to support the visualization of WHO’s system data through enabling submission via a single platform or through providing a specific function, rather than relying on comparatively limited data to address present inconsistencies. At the very least, platforms should provide an opportunity to compare data by individual specimen type, as evidenced by the observed variation in the isolate data in the WHO’s and ECDC’s platforms, despite reporting via a sophisticated platform using national data.

Although we were able to evaluate comparators, open-access platforms against all the available WHO’s system data, we acknowledge that some countries also engage in further closed surveillance networks (such as the Asian network for surveillance of resistant pathogens), semi-open access networks that look at a limited number of organisms (such as gram negative surveillance by the global study for monitoring antimicrobial resistant trends) or belong to networks that provide regular reports but have no interactive platform (Central Asian and European surveillance of antimicrobial resistance network). Our results raise concerns about the heterogeneity of the matched country data of some of the most established observatories. We recommend that those seeking to inform policy consider further evaluating the data held within these restricted-access networks. Our findings also reveal data discrepancies during the last full year of reporting before the COVID-19 pandemic, followed by a period of increased antimicrobial use and diverted laboratory capacity. These backdrops are highlighting a need to urgently improve data reliability across platforms to understand the true impact of the COVID-19 pandemic on global antimicrobial resistance. When evaluating the surveillance strategy in their specific regions, policy-makers should bear in mind that in some areas, current reporting capacity is likely to be more limited.

In conclusion, the surveillance data submitted to various supranational antimicrobial resistance monitoring platforms seem to be significantly heterogeneous, which may compromise their validity and undermine national and global strategies. This heterogeneity is particularly concerning for low- and middle-income countries as misinforming of their decision-makers may affect the perceived need for specific diagnostics or antimicrobial guidelines.

Policy-makers must be made aware of the potential unreliability of the platforms intended for informing strategy or outcomes. Mitigation measures must be taken to reduce surveillance bias through limited reporting and improve the ability to report more representative data in the short-term. These measures are particularly relevant in countries that need to improve their national surveillance platforms. Recent WHO recommendations to consider periodic strategic surveys in such circumstances seek to address this issue and may be further complimented if a minimum required data set is agreed on to streamline reporting and optimize representation in the short-term.
